# Risk practices for animal and human anthrax in Bangladesh: an exploratory study

**DOI:** 10.3402/iee.v3i0.21356

**Published:** 2013-11-27

**Authors:** Md. Saiful Islam, M. Jahangir Hossain, Andrea Mikolon, Shahana Parveen, M. Salah Uddin Khan, Najmul Haider, Apurba Chakraborty, Abu Mohammad Naser Titu, M. Waliur Rahman, Hossain M. S. Sazzad, Mahmudur Rahman, Emily S. Gurley, Stephen P. Luby

**Affiliations:** 1 Centre for Communicable Diseases, International Centre for Diarrhoeal Disease Research, Dhaka, Bangladesh; 2 Epidemiology Branch, Influenza Division, Centres for Disease Control and Prevention (CDC), Atlanta, GA, USA; 3 Institute of Epidemiology, Disease Control and Research, Dhaka, Bangladesh; 4 Global Disease Detection Branch, Division of Global Health Protection, Centres for Disease Control and Prevention (CDC), Atlanta, GA, USA

**Keywords:** anthrax, Bangladesh, ruminants, vulture, qualitative

## Abstract

**Introduction:**

From August 2009 to October 2010, International Centre for Diarrheal Disease Research, Bangladesh and the Institute of Epidemiology, Disease Control and Research together investigated 14 outbreaks of anthrax which included 140 animal and 273 human cases in 14 anthrax-affected villages. Our investigation objectives were to explore the context in which these outbreaks occurred, including livestock rearing practices, human handling of sick and dead animals, and the anthrax vaccination program.

**Methods:**

Field anthropologists used qualitative data-collection tools, including 15 hours of unstructured observations, 11 key informant interviews, 32 open-ended interviews, and 6 group discussions in 5 anthrax-affected villages.

**Results:**

Each cattle owner in the affected communities raised a median of six ruminants on their household premises. The ruminants were often grazed in pastures and fed supplementary rice straw, green grass, water hyacinth, rice husk, wheat bran, and oil cake; lactating cows were given dicalcium phosphate. Cattle represented a major financial investment. Since Islamic law forbids eating animals that die from natural causes, when anthrax-infected cattle were moribund, farmers often slaughtered them on the household premises while they were still alive so that the meat could be eaten. Farmers ate the meat and sold it to neighbors. Skinners removed and sold the hides from discarded carcasses. Farmers discarded the carcasses and slaughtering waste into ditches, bodies of water, or open fields. Cattle in the affected communities did not receive routine anthrax vaccine due to low production, poor distribution, and limited staffing for vaccination.

**Conclusion:**

Slaughtering anthrax-infected animals and disposing of butchering waste and carcasses in environments where ruminants live and graze, combined with limited vaccination, provided a context that permitted repeated anthrax outbreaks in animals and humans. Because of strong financial incentives, slaughtering moribund animals and discarding carcasses and waste products will likely continue. Long-term vaccination coverage for at-risk animal populations may reduce anthrax infection.

Anthrax is a zoonotic disease caused by spore-forming *Bacillus anthracis* (1, 2). Throughout the world, it causes illness in livestock, wildlife, and sometimes secondarily infects humans (3). The most common source of infection for ruminants is ingestion of spores during grazing in contaminated pastures, or through grass and water contaminated with anthrax spores (4–7). Domestic cattle, sheep, and goats can also become infected through concentrated feed that may include bone meal originating from anthrax-infected carcasses (8).

Anthrax infection is frequently fatal in ruminants. When the carcass of an anthrax-infected animal is cut open to obtain meat or the hide, the vegetative cells of anthrax are exposed to air and form spores (7). These spores can remain potent in the soil for several decades and may spread in the environment through scavenging birds, animals, and water (7, 9). Ongoing vaccination programs can break the cycle of transmission in domestic animals (4). Anthrax in livestock is frequently found in endemic areas where the veterinary public health infrastructure is weak and anthrax animal vaccination coverage is low (6).

In Bangladesh, anthrax is common among domestic ruminants (10). Since 1980s, researchers have reported 590 animals with laboratory-confirmed *B. anthracis* (10, 11). A few epidemiological studies were conducted during animal anthrax outbreaks between 1980 and 2010 in Bangladesh, but they were limited to quantitative investigations of individual-level risk factors (11–13). To develop a context-appropriate intervention for preventing animal infections and zoonotic transmission, we require an understanding of the broader context of these outbreaks that enable them to recur (14).

From August 2009 to October 2010, the Centre for Communicable Diseases under the International Centre for Diarrheal Disease Research, Bangladesh and the Institute of Epidemiology, Disease Control and Research investigated 14 outbreaks of anthrax in Bangladesh from 14 villages in three districts, which included 140 animal and 273 human cases of anthrax (13). A team of field anthropologists conducted exploratory investigations in five of these outbreaks in conjunction with the epidemiological outbreak investigations (13). The objectives of this article are to describe the context in which these outbreaks occurred, including livestock rearing practices, how owners managed sick and dead animals, and the anthrax vaccination program among outbreak communities.

## Methods

### Study site

**Fig. 1:**
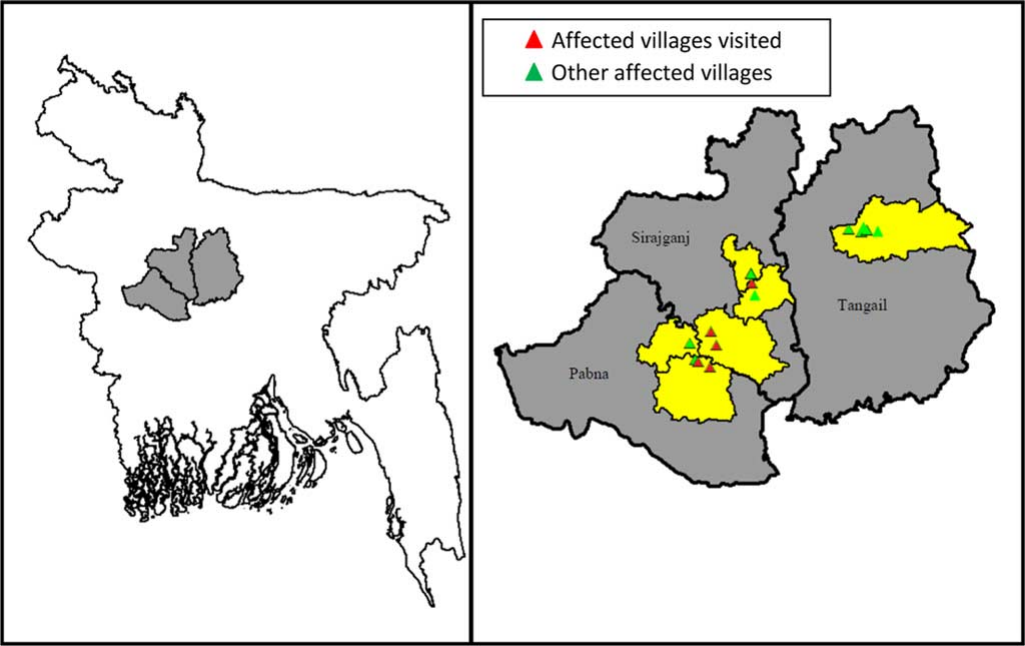
Outbreak districts and affected communities in Pabna, Sirajgonj and Tangail Districts, Bangladesh, 2009–2010.

We conducted this investigation in five anthrax-affected villages in four sub-districts: two villages in Santhia sub-district of Pabna District and one village in each of Shajadpur, Kamarkhand, and Belkuchi sub-districts of the Sirajgonj District in Bangladesh (Fig. 1). These four sub-districts are all low lying and flooded during the monsoon season every year. Approximately 1.5 million people live in these four sub-districts and 27% of residents aged 7 years and older have the ability to read and write; 93% of the residents are Muslim (15). In 2010, there were nine milk-processing companies operating in this region that provided a profitable market for milk. In 2010, there were approximately 1 million ruminants in these sub-districts, with 932 ruminants/km^2^, which is the highest ruminant density among sub-districts in Bangladesh (16), and the cattle in this area produced nearly half of the total milk products in Bangladesh (17).

### Study population, study design, and data collection

**Fig. 2:**
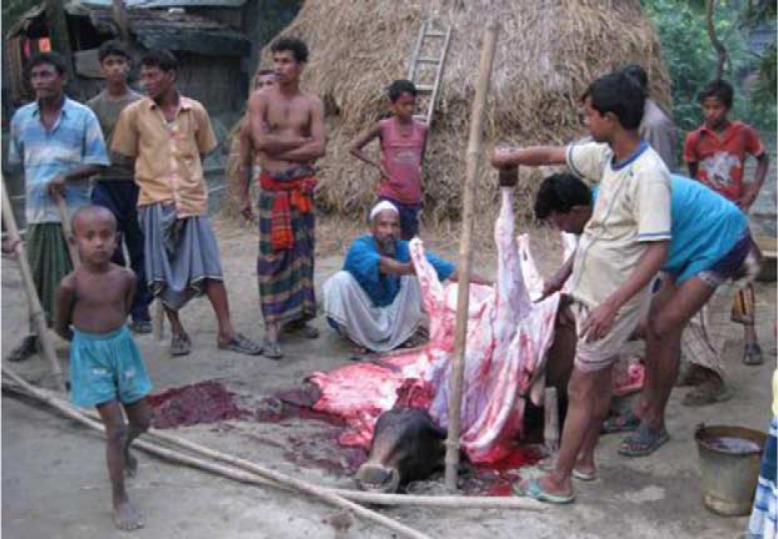
Butchering a cow on the back yard in front of the cowshed, Sirajgonj District, Bangladesh, 2010.

The team visited the five anthrax-affected villages and invited the village residents who participated in slaughtering anthrax-infected animals, the family members of the affected households, and the people who owned the sick and dead animals to participate in the study. We also invited the senior officials from the Livestock Research Institute, Dhaka, where the anthrax vaccine is produced in Bangladesh, the local livestock officers from the four affected sub-districts, oil cake producers (type of cattle feed made from nuts and seeds), and cattle feed store owners to participate in our study as key informants. We used unstructured observations, open-ended interviews (18, 19), group discussions, and key informant interviews to collect the qualitative data from October 2009 to September 2010. During visits to the villages, the field team conducted 15 hours of unstructured observation in the villages where animals had become sick, died, or were slaughtered during the outbreaks. The objectives of the unstructured observations were to identify the livestock feeding practices, slaughtering places, location of cowsheds, and places for discarding slaughtering waste and carcasses. The observations also helped build rapport with the community and select respondents for interviews. During data collection in an affected village, we found villagers slaughtering a sick cow and observed the cow being butchered (Fig. 2). Field anthropologists conducted 32 open-ended interviews to collect data on cattle rearing practices, available sources of feed for livestock, practices of slaughtering sick animals and discarding of dead animals, and supply and delivery of anthrax vaccine. The field team explored community norms for handling sick and dead animals, and discarding slaughtering waste and carcasses through six group discussions. The field team conducted 11 key informant interviews through which we explored the annual vaccine production and its distribution at the national level, the supply and delivery of anthrax vaccine to animals at the local level, and the sources of concentrated feedstuff in the affected villages. The team also reviewed the register books maintained by the livestock offices of each of the four affected sub-districts and collected information about the total number of livestock.

### Data analysis

The field team expanded the observation and interview field notes and reviewed them to identify themes. The data were then categorized according to the themes, translated into English, and typed into Microsoft Word. Within the themes, we compared the data that came from all research tools to verify and cross check our findings.

### Ethical considerations

The field team obtained informed verbal consent from respondents. This investigation was part of an emergency outbreak response, so a study protocol was not reviewed by a human subjects committee. However, the outbreak investigation methods were approved by the Government of Bangladesh.

## Findings

In 2009 and 2010, the reported practices of livestock feeding and managing sick and dead animals were consistent among all five outbreak-affected villages that we investigated. Most of the interviewed cattle owners earned their living from agriculture, and they mentioned that cattle rearing represented a major financial investment.

### Current livestock rearing practices

In the outbreak villages, ruminant owners raised a median of six ruminants (range 0–18) that included a median of five cattle and two goats. Owners kept their cattle in a cowshed and their goats either in their bedroom or on the veranda on their household premises. In the cowshed, the farmers fed the cattle dry rice straw, green leaves of rice, a variety of green grasses that grew locally, and napier grass (*Pennisetum purpureum*) gathered from the pastures. The farmers also reported feeding dicalcium phosphate (DCP) to lactating cows to increase their milk production. We identified 68 anthrax-infected ruminants, 29 of which were lactating cows, infected in the five villages during the outbreaks. In Bangladesh, June to October is the monsoon season, and all five outbreaks were reported during the monsoon season (20). In the beginning of the monsoon season, green leaves and tender stems grew from the old cut stems of rice in the pastures and farmers reported that they fed those tender stems to their livestock. During the investigation, we observed some villagers feeding water hyacinth to their cattle that had been collected from adjacent bodies of water. During the monsoon season, the pastures were flooded and the cattle were not taken to pastures. The high water level often carried water hyacinth near or inside the household premises, whereas in the dry season it remained confined to ponds and other bodies of water. Throughout the year, farmers also fed their cattle concentrated feed, including rice husks, wheat bran, and oil cakes which they purchased from the local markets. While exploring the concentrated feeds provided to the infected livestock, all the cattle owners reported that the feeds were locally produced, and to their knowledge the feed supplies did not include bone meal in their concentrates. The oil cake producers mentioned that they used mustard seeds, sesame seeds, linseed, and castor beans as raw materials for oil, and the oil cake was the by-product of extracting this oil. The farmers purchased the oil cakes locally to feed their cattle. The cattle feed shop owners and a local livestock officer from an affected sub-district said the rice husk and wheat bran were from local rice and flour mills.

### Slaughtering sick animals and disposal of butchering waste

Slaughtering moribund cattle was a common community practice. Because Islam forbids eating animals that die from natural causes, the farmers preferred slaughtering moribund cattle and goats while the cattle or goats were still alive so that the meat could be eaten. Therefore, when cattle or goats were on the verge of death, the cattle owners and their neighbors and friends often slaughtered the cattle to sell the meat in the community in an attempt to recoup some of the owners’ financial investment. One respondent explained that,

Cows and milk are the main income source for some families. If they (owners and their neighbours and friends) can slaughter a moribund cow, they can minimize their (owner's) financial loss to some extent by selling the meat.

The farmers slaughtered cattle in the yard of their household premises (Fig. 2). A few cattle were also slaughtered near or inside the cowshed, with one farmer reasoning that,

The cow was moribund and many of the family members and neighbours were not present to move the cow from the cowshed. So, we slaughtered the cow inside the cowshed.

In one village, the farmers also slaughtered a sick cow in the grazing field because they thought the animal would die before they could move it to the household.

When cattle were slaughtered on household premises, the farmers rinsed the slaughtering places with water to remove the blood. The animal owners usually threw the butchering waste in nearby ditches, bodies of water, or open fields. They mentioned that they often saw birds, dog, and foxes scavenging on the discarded butchering waste. Although slaughtering sick animals was common in the community, residents did not slaughter pregnant and very young animals also prohibited by Islam.

### Handling and disposal of carcasses

In the five anthrax-affected villages, when cattle died from illness, the owners often asked local skinners to take the skins and remove the carcasses away from the village to avoid the smell from decaying carcasses. The owners and the skinners took the carcasses either to the river bank or to agricultural fields. A usual practice was for 10–15 people to participate in removing one animal's skin, then one or two skinners would sell the skins in the leather market and split the profits among all the skinners. Cattle hides were sold for US$ 18–25 (Tk. 1500–2000), whereas goat or sheep hides were sold for US$ 1.2–1.8 (Tk. 100–150). Due to the very low price in the local market, skinners usually do not remove skins from dead goats and sheep.

The majority of the outbreaks occurred during the monsoon season when heavy rainfall occurred. Most of the cattle owners mentioned that they did not have dry land away from the household premises to bury the carcasses during the monsoon season, so they discarded the carcasses either in the flood waters or in the river. They said that the flow of the water usually took the carcasses away from the community. Villagers from these communities reported seeing carcasses, that had been discarded by villagers living upstream, floating in the river and flood waters. The participants from one village reported that they saw crows scavenging on the floating carcasses. In the village in Shajadpur sub-district, three owners buried their dead cattle on their household premises and one reported that even though he buried the carcass, his family members smelled a bad odor from the decaying carcass. Another farmer mentioned that when he buried the carcass, foxes and dogs dug it up and carried pieces to different locations where it rotted and smelled bad.

A group of local veterinary doctors, who had extensive involvement in visiting sick livestock and providing treatment in these affected communities, highlighted the role of vultures in scavenging on dead carcasses. However, many vultures in Bangladesh have died in recent years after consuming carcasses that have been treated with diclofenac sodium before death (21). Local veterinarians mentioned that the absence of vultures had increased the length of time that carcasses remained in the environment. A veterinary doctor who worked in a milk-processing factory for several years said,

Long ago when cattle died, the vultures would eat the carcass within a few hours or a day. Now no more vultures can be seen because of using diclofenac sodium in veterinary practice. As a result, the carcass remains on the ground for several days.

### Anthrax vaccination program

The officials of the Livestock Research Institute explained that the Government of Bangladesh has two laboratories that produce anthrax vaccine. Although these two laboratories have a target production of 5.1 million vaccine doses annually, their average annual vaccine production for the last 6 years (July 2004–June 2010) was 3.8 million doses, whereas the country's total ruminant population is approximately 48.7 million (22). The number of vaccine doses only covers a small fraction of the ruminant population. For example, in the four anthrax-affected sub-districts in this study, there were approximately 843,297 cattle and goats, yet only 46,000 doses of vaccine were distributed in the four sub-districts with anthrax-affected villages from July 2009 to June 2010. The principal scientific officer noted that only government livestock officers had the mandate to distribute and administer anthrax vaccine. In the four affected sub-districts, there were nine trained government vaccinators who were responsible for vaccinating livestock against anthrax, foot-and-mouth disease, hemorrhagic septicemia (*Pasteurella multocida*), and black quarter (*Clostridium chauvoei*). Due to limited staffing for anthrax vaccination, the local government livestock officers reported that they could not utilize the vaccine they received to vaccinate the susceptible ruminant population during the outbreaks. The livestock officers conducted a limited post-outbreak ring-vaccination strategy (the vaccination of all susceptible ruminants living in the affected community).

The local government livestock officers explained that an officer responsible for vaccination received a maximum travel allowance of approximately US$ 0.38 (Tk. 30) per day. This amount did not cover their cost to travel to distant villages, particularly in remote areas. Although the government set a reduced price of approximately US$ 0.67 (Tk. 50) for a vial of 100 doses of anthrax vaccine, the livestock officer unofficially charged the farmers US$0.13 (Tk. 10) extra for a single dose of vaccine to cover their additional expenses. The farmers often did not want to pay because they believed a government-supplied vaccine should be free of cost. Moreover, some farmers were concerned that vaccinating lactating cows would decrease milk production.

## Discussion

**Fig. 3:**
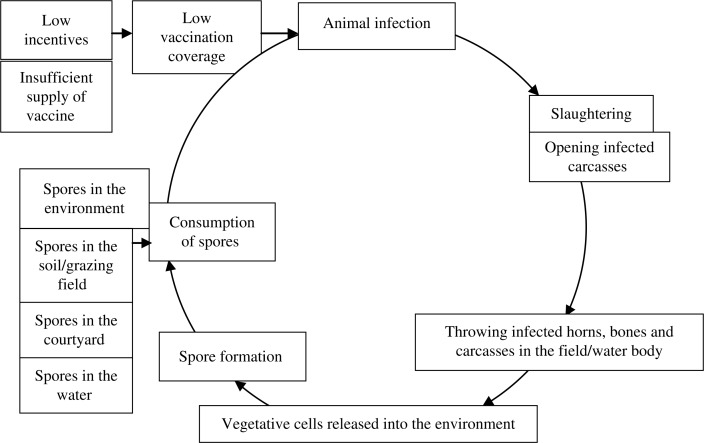
Possible cycle of animal infection that contributed to the persistence of anthrax spores in Pabna and Sirajgonj Districts, Bangladesh.

We have explored the factors that might put animals at risk of anthrax infection and how these factors may cyclically increase animal infection (Fig. 3). The slaughter of anthrax-infected animals and the disposal of butchering waste and carcasses in environments where ruminants live and graze, combined with limited vaccination, provided a context that permitted repeated anthrax outbreaks in animals and zoonotic transmission to humans. Steps to control anthrax should be aimed at breaking this cycle of infection (4).

Repeated outbreaks of anthrax among domestic ruminants have been reported in these outbreak districts since the 1980s which indicates anthrax spores are likely to be present in the environment (11). Anthrax spores can cause new infections when animals are exposed to contaminated soil or when animals graze or forage in contaminated environments (7, 23).

We have identified slaughtering anthrax-infected ruminants in the cowsheds, backyards, and household premises, where ruminants live could be potential sources of anthrax infection. As cattle represent a major financial investment (13), owners and their neighbors of slaughtered anthrax-infected animals and sold the meat in the community to recuperate some of their investment. Among Muslims, eating meat from animals that have died of natural causes is not permissible and only living ruminants are allowed to be slaughtered for food (24). Therefore, community members slaughtered sick ruminants as quickly as possible, with little consideration for where the slaughtering should be optimally performed. In a companion study, investigators found anthrax spores in the soil where anthrax-infected animals were slaughtered and in the animal bones that were found at the site (25), suggesting that spores stay viable in the environment where anthrax-infected ruminants were slaughtered and where dead animals or their carcasses have been discarded.

Opening anthrax-infected carcass for hides and discarding them in the environment permit the vegetative cells of *B*. *anthracis* to form spores and contaminate the environment (7). In outbreak areas, flooding during the monsoon season limits the ability to bury carcasses. In flood-affected areas, the global recommendation for anthrax control emphasizes burning carcasses (4). Bangladesh lacks facilities for incineration and for sophisticated carcass processing. Moreover, burning carcasses might not be economically feasible to low-income rural populations because of associated fuel costs. World Health Organization and the Centres for Disease Control and Prevention recommend not opening carcasses of animals that have died from suspected anthrax (6, 26). Since *B. anthracis* is an aerobic bacterium, the putrefactive process can kill the vegetative cells of the bacteria in an unopened carcass within 48–72 hours of death (23, 27). However, this recommendation is not frequently followed in Bangladesh due to the economic incentives of selling hides. Skinners take the opportunity to recover hides from the discarded carcasses because local health inspectors do not monitor carcass disposal.

Anthrax outbreaks among animals have frequently occurred in pastures where a common source of infection is grazing on grasses grown in soil contaminated with anthrax spores, which may retain their infectivity for many years (4, 6, 23). Moreover, rainwater may collect and gather spores in low-lying pastures, and contaminate fresh feed such as new grass and green rice straw (23). In the outbreak communities, the most common sources of feed for the infected cattle were grasses and straw grown in local pastures. Farmers graze cattle in low-lying pastures which flood every year to reduce the need to purchase supplementary feed for their cattle and therefore minimize their production cost. In the outbreak communities, another source of fresh feed for cattle was water hyacinth from adjacent bodies of water. Because anthrax spores have high surface hydrophobicity, the water hyacinths are also likely to be contaminated with floating anthrax spores that have been carried downstream and concentrated in the low-lying bodies of water (23, 28, 29).

Another potential source of anthrax could have been concentrated feed produced from anthrax-infected carcasses. In Bangladesh, there are no rendering plants that produce bone meal (30), and locally produced rice husks, wheat bran, and oil cakes are unlikely to include bone meal. However, Bangladesh imports ruminant by-products from Europe and the DCP provided to lactating cows may have animal by-product (30–32). However, it is notable that the anthrax outbreaks occurred only during the monsoon season and feeding DCP was a year-round activity. Therefore it is less likely that the DCP was the source of these anthrax outbreaks in Bangladesh. Further describing the production chain of DCP and assessing the association of DCP with animal anthrax cases in outbreak investigations may provide further insight on its potential role.

An insufficient supply of anthrax vaccine, lack of staffing for vaccination, and an ineffective vaccine strategy that fails to target the highest risk areas, has left livestock susceptible to anthrax infection. Although the official price per dose of anthrax vaccine is low, a post-outbreak ring-vaccination strategy is often implemented in high risk areas due to staffing shortages. However, the live-attenuated vaccine can only protect an animal effectively for 6–9 months; therefore, vaccinated cattle are susceptible again during the next year (4, 33). In addition, livestock officers are reluctant to visit low-income communities and remote rural areas that are at risk due to the insufficient travel allowances for vaccination.

Vultures play a crucial role to the overall health of the ecosystem through scavenging on discarded carcasses (34). Although some have argued that vultures may contribute to the transmission of anthrax by contaminating water holes during washing their beaks and feathers, there is no scientific evidence of this assumption (34). Vegetative cells of anthrax are fragile and die off quickly in the water. Even, if some vegetative cells of anthrax form spores, the small number of residual anthrax organism on the feet, beaks, and feathers or in the guts of vultures are not sufficient to initiate new animal infection (35).

Vultures reduce the spread of anthrax by consuming the bacilli-laden soft tissues of anthrax-infected carcasses before the vegetative anthrax cells turn into spores (34, 36, 37). Large numbers of vultures increase carcass consumption. The decline of Asian vultures may have increased the incidence of diseases such as rabies and anthrax (38). Since 1990's, three species of vultures have declined by more than 97% in South Asia and these species are now rare in Bangladesh due to the wide use of diclofenac sodium in veterinary practice (21, 39, 40). Although the government of Bangladesh banned veterinary diclofenac, it is still widely used throughout the country (41). In the absence of rapidly scavenging vultures, carcasses remain exposed for several days, allowing dogs, foxes, crows, and flies to spread the vegetative cells of anthrax, which then form spores within several hours after exposure to air, contributing to the cycle of anthrax transmission (7, 42).

An important limitation of this investigation was that we only investigated outbreaks occurring in low-lying areas, although anthrax outbreaks have also been reported in other geographical areas of Bangladesh. However, we found that the practices of rearing livestock and slaughtering sick cattle in the affected low-lying areas were largely similar to the practices found in recent outbreak investigations conducted in other areas of Bangladesh (Aushraful Islam, Meherpur anthrax outbreak investigation 2011, personal communication).

For anthrax control, one strategy could be to limit animal exposure to contaminated environments and restrict fresh animal feed collected from those environments. Our findings and previous literature suggest that the household premises and pastures of the communities that had experienced anthrax outbreaks in recent years might have already been contaminated with anthrax spores. Restricting animal grazing practices will increase the ruminant production cost by requiring more feed and is therefore likely to be unacceptable to low-income rural Bangladeshi farmers. Another control strategy could be to prevent recontamination of the environment through the safe disposal of anthrax-infected carcasses. However, with the social norm of slaughtering sick ruminants for consumption and selling their hides, and the economic reality of low-income rural Bangladeshis, interventions solely focused on changing behaviors related to slaughtering sick animals and the disposal of anthrax-infected carcasses in the environment, without economic incentives, are unlikely to be effective. Options for initiating livestock insurance as a risk management practice can be explored. Various models of livestock insurance have been successfully used to control animal disease outbreaks in other countries leading to disease eradication (43). Considering the economic impact of animal anthrax, an insurance strategy might be acceptable and feasible for individual cattle raisers. Piloting various models of livestock insurance in affected sub-districts can explore the feasibility, scalability, and sustainability of using livestock insurance as a method to increase disease reporting and control.

Because a majority of the outbreaks occurred during the monsoon season, animal vaccination just before the monsoon season in areas that have experienced recent anthrax outbreaks may reduce the risk of further outbreaks (4). The scarcity of vaccines and limited vaccination staff are a barrier to anthrax vaccination coverage. Bangladesh has two anthrax vaccine laboratories with an annual production of around 4 million doses. This amount of vaccines may be sufficient if used mainly for the susceptible animal population in Bangladesh. Research to explore strategies to make this vaccine available in at-risk areas could increase vaccination coverage. Micro-planning, a community-based process which enables local residents to prepare and implement programs, has proven to be a successful approach for the Extended Program on Immunization in Bangladesh to increase vaccination coverage in rural and remote areas (44). Research to pilot such an approach in some sub-districts of Pabna and Sirajgonj Districts could also increase animal anthrax vaccination coverage. The privatization of the fertilizer sector in Bangladesh resulted in a threefold increase of fertilizer use over a 10-year period (45). To increase the animal anthrax vaccination coverage, options for involving the private sector in providing anthrax vaccine should be explored.

To promote the important role that vultures play in the ecosystem through scavenging on carcasses, the Ministry of Livestock and Fisheries, the Ministry of Forest and Environment, and public health professionals working in veterinary and human health sectors should work together to identify appropriate steps for vulture conservation. Following the ban on diclofenac, several neighboring countries successfully promoted alternative drugs to reduce diclofenac use in veterinary practice (46). Piloting the acceptability and effectiveness of alternative veterinary anti-inflammatory drugs in Bangladesh should be explored. Moreover, increasing awareness related to the devastating impact of diclofenac sodium on vultures among farmers, veterinary and public health professionals, and government officials might help to sensitize on the plight of vultures and to limit the use of veterinary diclofenac (46).
